# Insights into the function of cytoglobin

**DOI:** 10.1042/BST20230081

**Published:** 2023-09-18

**Authors:** Brandon J. Reeder

**Affiliations:** School of Life Sciences, University of Essex, Wivenhoe Park, Colchester, Essex, U.K.

**Keywords:** cytoglobin, disulphide bonds, protein structure, redox chemistry

## Abstract

Since its discovery in 2001, the function of cytoglobin has remained elusive. Through extensive *in vitro* and *in vivo* research, a range of potential physiological and pathological mechanisms has emerged for this multifunctional member of the hemoglobin family. Currently, over 200 research publications have examined different aspects of cytoglobin structure, redox chemistry and potential roles in cell signalling pathways. This research is wide ranging, but common themes have emerged throughout the research. This review examines the current structural, biochemical and *in vivo* knowledge of cytoglobin published over the past two decades. Radical scavenging, nitric oxide homeostasis, lipid binding and oxidation and the role of an intramolecular disulfide bond on the redox chemistry are examined, together with aspects and roles for Cygb in cancer progression and liver fibrosis.

## Introduction

Cytoglobin (Cygb) is the fourth member of the globin family found in humans after erythrocyte haemoglobin (Hb), myoglobin (Mb) and neuroglobin (Ngb). Discovered in 2001 and initially named stellate cell activating protein (STAP) [1] and histoglobin [2] the physiological function of this protein still remains to be elucidated. As a trivia side note, an episode of Star Trek Voyager (season 7 episode 5, ‘Critical Care'), first aired in late 2000, used the term cytoglobin as a fictional drug that was used to treat ‘chromoviral infections and arterial aging' [3]. The name of the real-life protein is derived from its ubiquitous expression of the globin in vertebrate tissue [4]. Cygb expression is in the cytoplasm of a wide variety of cell types including fibroblasts, chondroblasts, osteoblasts, and hepatic stellate cells (HSCs) [5]. Cygb has also been located in the nuclei of numerous cell types including neurons [5,6], the solitary tract [7], some hepatocytes [8] and myogenic progenitor cells [9]. It is the ubiquitous nature of the protein that gives us an indication of why it has been more difficult than most to assign a primary cellular role. Other globins, unlike Cygb, are typically expressed in a small range of specific tissues or cell types, hence establishing their primary role(s) within a cell has been somewhat more straightforward. Hb, one of the most extensively studied of all proteins, is expressed in erythroblasts for oxygen transport in erythrocytes. Mb is expressed predominantly in the myocytes to facilitate oxygen diffusion between the blood vessels and the myocyte mitochondria [10] or to store oxygen, particularly in diving animals [11,12]. Ngb is predominantly expressed in nerve cells [13] where it acts as a nitric oxide (NO) regulator [14], protecting neurons from hypoxic-ischaemic insults [5]. Each of these globins has confirmed or proposed secondary functions that typically involve the redox chemistry of the protein. Androglobin (Adgb), the fifth globin discovered in 2012, is primarily expressed in ciliated cells and spermatocytes, although studies into the structure of this multi-domain, circularly permuted protein are ongoing and its function is currently elusive [15,16].

Phylogenetic analysis of Cygb suggests a common ancestor with vertebrate Mb ∼500 Mya [4] and is also related to the eye globin (GbE) of avians with a common ancestor ∼300 Mya [17]. However, it has also been speculated that Cygb may be more closely related to the Hbs of Agnatha (jawless fish), with Mb being a distinct branch [17]. In teleost fish, Cygb appears to have been duplicated ∼320–350 Mya with two paralogous Cygb genes Cygb1 and Cygb2 [18]. Both proteins are expressed in a range of tissues, however, Cygb2 exhibits high expression in neuronal tissues, suggesting that the two globins have sub-functionalisation following gene duplication [18].

## Cytoglobin structure and key functional amino acids

**Figure 1.**
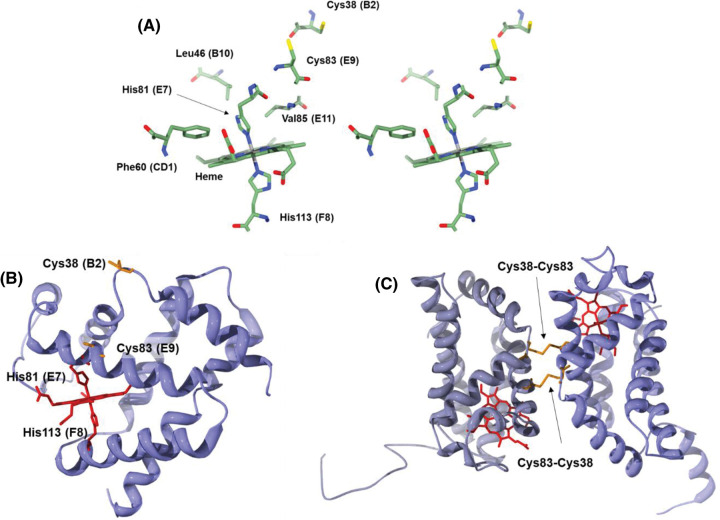
Structure of cytoglobin. (**A**) Stereoview of the heme pocket environment with notable structural and functional amino acids (PDB: 2DC3). The heme is shown edge-on with the heme pocket entrance to the front. (**B**) Monomeric protein, without intramolecular disulfide bond (PDB: 1VH5). The heme (red) is shown edge-on with the heme pocket entrance to the left. Proximal (His 113) and distal (His 81) are shown in red and cysteines in orange. (**C**) Dimer conformation showing an intermolecular disulfide bond (Cys38–Cys83, PDB: 2DC3). Part of the C-terminal extension is seen in the lower left side and part of the N-terminal extension showing alpha helical structure is seen in the lower right due to crystal packing. Heme is in red stick and cysteines in orange. SwissPDB viewer v4.1.0 and POV-Ray v3.7.0 was used to create the figures.

The structure of cytoglobin shows the typical 3-over-3 alpha helical fold of non-truncated globins [19]. The heme iron is hexacoordinate with a bis-His ligand set, similar to that of Ngb and some phytoglobins (PhytoGb — previously non-symbiotic globins), but unlike the pentacoordinate geometry of respiratory globins such as Mb and Hb. Hexacoordinate globins typically show enhanced thermal stability. The thermal stability of human Cygb is high with a melting temperature of 95°C, just slightly below that of Ngb [20]. Highly conserved residues in the heme pocket are similar to those of other globins including the distal His81(E7) and proximal His113(F8) histidine pair, a Phe(CD1) to stabilise heme binding and Leu46(B10) plus Val85(E11) for steric control of ligand binding and heme pocket cavity arrangement [21–23] (Figure 1A). The protein can exist in three conformations *in vitro* depending on the redox state of two cysteines, Cys38(B2) and Cys83(E9). Various crystal structures show the protein in either a monomeric state with the cysteines reduced (monomer_SH_) or mutated (e.g. PDB:1VH5 [24] Figure 1B), or a dimer with two intermolecular disulfide bonds, Cys38–Cys83 and Cys 83–38 (dimer_S–S_) (e.g. PDB: 2DC3 [25], Figure 1C). A third state, yet to be crystallised, is that of a monomer with an intramolecular disulfide (monomer_S–S_).

Cygb has extended N- and C-terminal extensions beyond the typical alpha helical fold of the globular part of the protein with each extension ∼20 amino acids long, making the human protein 190 amino acids in length and a molecular weight of ∼21 kDa [4]. Crystal structures have shown that these extended domains are primarily disordered, however, structural predictions (e.g. Alphafold [26]) and some crystal structures show alpha helical structure for the N-terminal extension (e.g. PDB: 2DC3 [25]). Truncating the protein to remove the extended N- and C-termini results in a slight decrease in thermal and structural stability [27]. Truncation decreases the superoxide-scavenging capability (C-terminal loss only) and influences the monomer_S–S_ conformation. The Cygb-1 from teleost fish are typically truncated, having lost the C-terminal intron. The N-terminal extension is absent from zebrafish Cygb-2, possibly by a mutation in the lineage of *D. rerio* [18]. Globin X (GbX) exhibits extended N- and C-terminal extensions, like that of Cygb [28]. However, unlike Cygb the N-terminus of GbX has conserved myristoylation and palmitoylation sites anchoring GbX to the cell membrane [29].

There has been considerable debate over the oligomeric state of the protein *in vivo*. This is a critical question to be answered as the biochemical and biophysical properties of the monomer_S–S_ form behaves very differently from the other two states. The protein monomer_S–S_ form has a distal histidine off rate over 600-fold faster than the monomer_SH_ or dimer_S–S_, with only slight changes to the distal histidine on rate [30]. Therefore, in the monomer_S–S_ state the protein has more pentacoordinate-like features, as exhibited by faster rates of exogenous ligand binding and higher affinities [31]. Allosteric ligand binding has also been reported in the dimeric protein [32,33]. Initial size exclusion chromatography (SEC) analysis of the protein, which is based on the hydrodynamic radius of the protein, suggests a primarily dimeric form of the protein [34]. However, mass spectrometric analysis of Cygb suggests that the B2 and E9 cysteines of Cygb form intramolecular disulfide bonds rather than intermolecular bonds and that dimerisation is not based on the presence of the intermolecular disulfide bonds [35]. It is interesting to note that mutants of the distal E7 histidine result in primarily monomeric proteins by SEC analysis, unlike the wild-type protein [36]. It has also been observed that the hydrodynamic diameter of Cygb deviates from other globins due to the N- and C-terminal extensions [37]. Analysis by SEC-MALLS (Multi-Angle Laser Light Scattering), which does not require reliable molar mass markers, shows the protein as a monomer and a truncated form of the protein without the N- and C-terminal extension also shows a monomer by standard SEC analysis [37]. Therefore, the current evidence supports Cygb as monomer in solution and in the range of concentrations expected *in vivo*.

## Cytoglobin redox chemistry

Cygb shows several activities that could have potential physiological or pathological roles. Cygb binds oxygen with an affinity of 0.2–3 Torr, depending on the conformation of the protein and the oxidation state of the disulfide bond [37,38]. This is in the same range as the oxygen affinity of Mb. As Cygb is closely related to Mb, an oxygen-carrying role was proposed in early studies on the globin [13,39]. Other studies also support a role of Cygb in O_2_ homeostasis, with disruption of O_2_ homeostasis activating HSCs in liver tissues inducing hepatic fibrogenesis [38,40]. However, expression level studies of the intra-retinal distribution of Cygb in the murine eye suggests that Cygb is unlikely to have a role as a respiratory oxygen carrier [41]. Additionally, the concentration of Cygb in most cells is in the low micromolar range, hence Cygb oxygen carrying would have little direct impact on mitochondrial respiration [33].

As with all globins, oxyferrous Cygb shows rapid nitric oxide dioxygenase (NOD) activity, converting NO to nitrate with the oxidation of the Cygb from ferrous to ferric. A rate constant of 3 × 10^7^ M^−1^ s^−1^ for this reactivity [42] is only slightly lower than that for other globins such as Ngb and Hb [43]. Numerous *in vitro* and *in vivo* studies suggest a physiological or pathological role for this redox chemistry (currently 39 articles). Such studies show that an enhanced expression of Cygb in fibroblasts resulted in decreased NO consumption and intracellular nitrate production [44]. Furthermore, Cygb deficiency is thought to hinder NOD activity, resulting in NO diffusion from HSC cells to neighbouring hepatate cells, leading to mitochondrial toxicity [45]. The cysteine oxidation state has a moderate, 4-fold, effect on NOD activity [46]. In Cygb(−/−) mice, the NOD function of Cygb is thought to play a critical role in regulating blood pressure and vascular tone [47].

Under hypoxic conditions, Cygb also shows a high nitrite reductase (NiR) activity, generating NO from nitrite. Typical NiR rate constants for globins range from 0.12 M^−1^ s^−1^ for Hb (T state) and Ngb [48,49] to 26.7 M^−1^ s^−1^ for globin X [50]. Cygb shows a similar range from 0.26 and 0.63 M^−1^ for the monomer_S-H_ and dimer_S–S_, respectively, to 32.3 M^−1^ s^−1^ for the monomer_S–S_ [51]. Thus with an activity range of over two orders of magnitude, cysteine oxidation could act as a molecular redox switch under oxidative conditions [51]. Ligand migration pathways are also affected by this proposed cysteine oxidation molecular switch [52]. It should be noted though, that while the B2 cysteine is highly conserved, the E9 cysteine is not. Therefore, if the B2–E9 cysteine oxidation does play a role in functional molecular switching, then this is an adaptation that requires further phylogenetic and biochemical study. Intramolecular disulfide formation is induced with small amounts of peroxide at low oxygen, making Cygb physiologically sensitive to low fluxes of peroxide under hypoxic conditions [32]. By enhancing the histidine off rate and opening the heme pocket, such cysteine oxidation would significantly enhance NiR activity under conditions of hypoxic stress. Such enhancement is not observed at normoxic conditions, indicating that the formation of alternate oxygenated thiol intermediates may occur [32]. The NiR activity, although many orders of magnitude slower than NOD activity, has been proposed to play an important role in NO generation and soluble guanylyl cyclase activation [53]. Several studies point to a role of Cygb in detoxifying reactive oxygen species (ROS). Some of the findings include Cygb attenuating pancreatic cancer growth via scavenging ROS [54], attenuation of ROS mediated liver fibrosis [55] and correlation with Cygb and Ngb with ROS scavenging in mice [56].

For such redox activities to be physiologically relevant there needs to be an effective reducing mechanism to return the high oxidation states of Cygb to ferrous *in vivo*. *In vitro* ferric Cygb reacts rapidly with ascorbate, two orders of magnitude faster than pentacoordinate globins such as Mb and Hb [57]. In addition, cytochrome b5/cytochrome b5 reductase reduces ferric Cygb 250-fold faster than Hb and Mb and up to 100-fold faster than 5 mM ascorbate [58]. The rapid *in vivo* reduction systems of Cygb endorses redox activity as a physiological role, such NO metabolism [59].

## Role of cytoglobin in cancer and fibrosis

The link between Cygb and cancer progression, particularly with hypoxic tumours, is well established from numerous studies on a variety of cancer types. Table 1 summarises the major finding of these studies. The consensus is that Cygb can function as a tumour suppressor, frequently correlating with the down-regulation in human cancers under conditions of normoxia [60–62]. However, under conditions of hypoxia, up-regulation of Cygb is often observed in solid tumours. A number of transcription factors are involved in hypoxia-induced Cygb up-regulation. Hypoxia inducing factor (HIF-1α) up-regulation of Cygb has been reported by numerous studies [63–68]. In addition, inhibition of calcineurin, NFAT, and/or AP-1 activities have been shown to decrease endogenous Cygb transcription in myocytes [66]. Solid neoplasms typically show decreased oxygen supply, leading to changes in cell morphology [69]. This frequently leads to resistance of hypoxic tumours to radiotherapy and some chemotherapeutic agents [70,71]. Improved outcomes of radiation therapy have been noted with oxygen therapy [60,72]. It has been proposed, therefore, that Cygb is bimodal, being a tumour suppressor under conditions of normoxia, but behaves to promote tumour growth under conditions of hypoxia [73].

**Table 1:** Observed links between cytoglobin and cancer development or effects of cytoglobin overexpression/silencing/knockdown on cancer progression

Cancer/cell line	Cytoglobin expression	Significant findings and conclusions	Date of study	Reference
Early stage squamous cell oesophageal cancer	Down-regulated	Cygb down-regulated typically by ∼70%. Promotor hypermethylation.	2006	[61]
Pulmonary tumour cells (A549)	Up-regulated (hypoxia)	Correlation between Cygb up-regulation and hypoxia.	2006	[74]
Oral squamous cell carcinoma	Up-regulated	65% Cygb promotor methylation observed.	2006	[75]
Lung cancer	Down-regulated	Implication of Cygb in the pathogenesis of non-small cell lung cancer.	2006	[62]
Lung and breast cancer cell line	Down-regulated/knockdown	Cygb promotor methylation correlates with gene silencing. Cygb knockdown increased colony formation.	2008	[76]
Head and neck squamous cell carcinoma	Down-regulated	Cygb mRNA expression showed a correlation with tumour hypoxia.	2009	[77]
Human glioblastoma multiforme cell lines, human primary tumour specimens	Up-regulated (hypoxia)	Up-regulated in ductal cells following 48 h hypoxia.	2010	[78]
Breast and lung tumours	Down-regulated	Cygb correlated with hypoxia.	2011	[79]
Mouse model.	Knockout	Drug-induced (DEN) liver and lung tumours in knockout mice.	2011	[80]
Oesophageal cancer cells	Down-regulated	Protection from chemically induced oxidative stress (non-physiological concentrations).	2012	[81]
Non-small cell lung cancer cell lines	Down-regulated	Cygb promoter was methylated in 64% of cell lines. Demethylation partially restored Cygb expression in cell lines.	2013	[73]
Non-small cell lung cancer cell lines	Overexpressed	Reduction in cell tumourigenicity, diminished migratory potential.	2013	[73]
Gliomas	Loss	Cygb loss may contribute to tumour recurrence and a worse prognosis in gliomas.	2013	[82]
Archived ovarian cancer specimens	Down (62% of samples)	Cygb down-regulation positively correlated with advanced FIGO stage and tumour grade.	2014	[83]
SKOV3/SW626 cell lines	Overexpressed/silenced	Overexpression inhibited cell growth, invasion, cell cycle progression and cyclin D1 expression, silencing promoted cell proliferation, invasion, cell cycle transition and cyclin D1 expression.	2014	[83]
Mouse NASH model (choline-deficient amino acid-defined diet)	Knockout	Prominent inflammation and fibrosis and liver cancer in Cygb (−/−) mice. Ameliorated by N-acetyl cysteine treatment.	2015	[84]
Primary neonatal human epidermal keratinocytes and MCF7 cells	Down-regulated (HEKn) Overexpressed/silenced (MCF7)	Direct regulation of CYGB by ΔNp63α. CYGB has a protective role in proliferating keratinocytes +/− H_2_O_2_ treatment.	2016	[85]
Mouse model	Knockout	Increased colonic inflammation and macroscopic tumour development in knockout mice. Neurexophilin and PC-esterase domain family member 4 (Nxpe4) also decreased.	2018	[86]
Breast cancer MDA-MB-468 cells	Induced (5F 203)/silenced	Caspases and CYGB promote 5F 203-mediated apoptosis. Silencing CYGB attenuated the ability of 5F 203 to induce caspase-3/-7 activation, proapoptotic gene expression, LMP, and cathepsin B release in MDA-MB-468 cells.	2019	[87]
Oral squamous cell carcinoma PE/CA-PJ41	Overexpressed	Increased cell growth and motility. Cygb up regulates apoptosis regulating cardiolipin and protects cells from *cis*-platin-mediated oxidative stress.	2021	[88]
HCT116 and SW620 human CRC cells	Overexpressed/silenced	Cygb overexpression increased lipid ROS and malondialdehyde accumulation, disrupted mitochondrial function. Correlation between Cygb expression and key genes YAP1 and ACSL4 in ferroptosis pathway.	2021	[89]
Pancreatic cancer and transgenic mice	Knockout	Cygb negatively correlated with tumour size. Cygb suppresses pancreatic stellate cell activation, pancreatic fibrosis, and tumour growth.	2022	[54]

The cellular pathways involving Cygb in cancer suppression or promotion are still unclear. However, up-regulation of the NFkappaB/iNOS signal pathway and NO production has been observed in human cardiac stem/progenitor cells with up-regulated Cygb, suggesting Cygb functions as a pro-survival factor in response to oxidative stress [90]. Furthermore, in melanocytes knockdown or overexpression of Cygb sensitised or protected the cells against ROS and reactive nitrogen species (RNS) induced apoptosis, respectively, with Cygb enhancing heme oxygenase-1 and NRF2 expression [91]. CYGB gene is regulated by both promoter methylation and tumour hypoxia in head and neck squamous cell carcinomas with expression correlating with the tumour's biological aggression [77]. Current knowledge of the behaviour of Cygb in tumours suggests that expression of Cygb could be used as a biomarker for early detection of cancer or liver fibrosis [91–93]. Previous reviews have examined the role of Cygb in cancer in more detail, focusing on its role in hypoxia [60] or as a biomarker for diagnosis and management [93]. Importantly, Cygb overexpression, as observed with some other globins, arrests cell cycling at the G1 phase, resulting in impaired cell proliferation, possibly by hyperphosphorylation of tumour suppressor retinoblastoma protein [94]. Furthermore, S phase kinase-associated protein 2, a highly oncogenic protein that triggers G1/S transition of cell cycle, has been shown to interact with Cygb and facilitate the periodic degradation of Cygb, promoting cell cycle progression [94].

Cygb, originally discovered in hepatocytes as STAP, is up-regulated in hepatic fibrosis. Cygb overexpression reduces tissue fibrosis in both toxic and cholestatic models of liver injury and promotes recovery after the onset of injury [95]. Carbon tetrachloride challenge induces liver fibrosis with early (24 h) induction of Cygb in fibroblasts of various tissues and hepatic HSCs, 24 h before the type I collagen Col1a1 gene [92]. Cygb expression levels may therefore act as a sensitive early marker for liver fibrosis and is postulated to play a role in collagen metabolism [92]. Furthermore, Cygb may induce protection via apoptosis as reported in thioacetamide-induced liver fibrosis in expression in rats, Cygb-transgenic mice and human stellate cells. Mechanisms of this protection may include activation of caspase cascade pathways, acting as a scavenger of RNS and ROS, as well as a potent regulator of HSC cell activation [96–98].

## Lipid binding, lipid oxidation and cell signalling

Unusual amongst vertebrate globins, Cygb interacts with lipids and fatty acids resulting in marked changes to the heme geometry and redox activity [99]. Only the monomer_S–S_ form exhibits this conformational change and only in the ferric oxidation state [99]. Interaction with oleate, cardiolipin or similar lipids and fatty acids leads to a change in heme coordination from hexacoordinate to pentacoordinate state [100] with such changes in the heme coordination occurring at ∼1 : 1 lipid:protein ratio [99]. Similar interactions with lipid or lipid-based molecules typically require an excess to induce a structural change. For example, the interaction of cytochrome c with cardiolipin requires ∼30 cardiolipin molecules per protein to allow maximum change from hexa- to penta-coordination, as measured by the ability to bind ligands such as CO to the heme iron [101]. Current evidence suggests that there is a specific site of binding between the lipid and Cygb. Although no crystal structures of a lipid-bound Cygb have yet been forthcoming, binding models and mutagenesis studies have given insights into the likely position of lipid binding. Lipid binding models of oleic acid, DOPA (dioleyl phosphatidic acid (1,2-dioleoyl-*sn*-glycero-3-phosphate) and PIP3 (dioleyl phosphatidylinositol 3,4,5-trisphosphate (1,2-dioleoyl-*sn*-glycero-3-phosphoinositol 3,4,5-trisphosphate) predicts the aliphatic chain extending into the hydrophobic core of the protein, with phosphate groups interacting with surface amino acids such as Lys111 and Lys116 and the ester groups interacting with Arg84 and Thr91 [102]. Mutation of Arg84(E10) and Lys116(F10) in particular lead to significant decreases in lipid binding affinity and are surface exposed and close to the entrance of the heme pocket [103].

It has been demonstrated that Cygb is also an effective lipid peroxidase *in vitro*, with the monomer_S–S_ form having significantly higher levels of activity compared with the monomer_SH_ and dimer_S–S_ states [100]. Anionic phospholipids are oxidised in the presence of peroxide, but these phospholipids can also enhance the peroxidatic activity of Cygb [102]. These lipid oxidation reactions are more rapid compared with pentacoordinate respiratory globins, but Ngb, another hexacoordinate globin shows no lipid peroxidase activity under similar conditions [99]. Both Mb and Hb are known or proposed to contribute to lipid oxidation reactions under pathological conditions such as vasospasm following intracranial hemorrhage [104–106] or acute kidney injury following rhabdomyolysis [106–109]. The mechanisms for these involve the formation of radical-based lipids from peroxide-driven ferric-ferryl globin redox chemistry that can result in a cascade of lipid oxidation. The resulting lipid oxidation products frequently show potent cell signalling properties such as vasoactivity [110,111] or activating the electrophile responsive element [112]. For more in-depth mechanisms and implications of these globin-generated lipid oxidation products see previous reviews [113–116].

It has been observed that Cygb overexpression in rat hepatic stellate cells decreases lipid radicals as shown by decreased levels of lipid peroxidation biomarkers malondialdehyde and 4-hydroxy-2-nonenal [95]. In a THP-1 acute monocytic leukaemia human cell line, Cygb addition significantly attenuated the effects of oxidised low density lipoproteins (LDL) [117]. Furthermore, injection of recombinant Cygb into atherosclerotic rats significantly decreased LDL-cholesterol and increased HDL-cholesterol, inferring that Cygb may be able to prevent atherosclerosis by regulating lipid metabolism and oxidative stress [117]. This apparent dichotomy in the *in vitro* observations of prevalent lipid oxidation with cell responses may be explained by the observation that lipid peroxidation, either through enzymatic or non-enzymatic pathways, results in a conversion of the saturated fatty acid to an oxylipidome. Many members of this oxylipidome are electrophilic and can modulate antioxidant and anti-inflammatory protective pathways [118]. Hence, lipid oxidation reactions up-regulate the antioxidant defence or induce apoptosis [112,119]. Therefore, it is reasonable to suggest that the lipid peroxidase activity of Cygb forms oxygenated lipid mediators that induce protective cellular responses *in vivo* [100,102]. However, further studies are required to identify and confirm such pathways. A recent study has demonstrated that hydrogen peroxide and NADPH oxidase 4 (NOX4) induced the import of Cygb into the nucleus of vascular smooth muscle cells [120]. The data supports a new potential function of Cygb to inhibit DNA damage and regulation of genes in the vasculature though binding with HMGB2, a non-histone DNA interacting protein. Such subcellular localisation of Cygb under oxidative conditions has interesting implications for cancer research and lipid signalling in the nucleus.

## Summary

**Figure 2.**
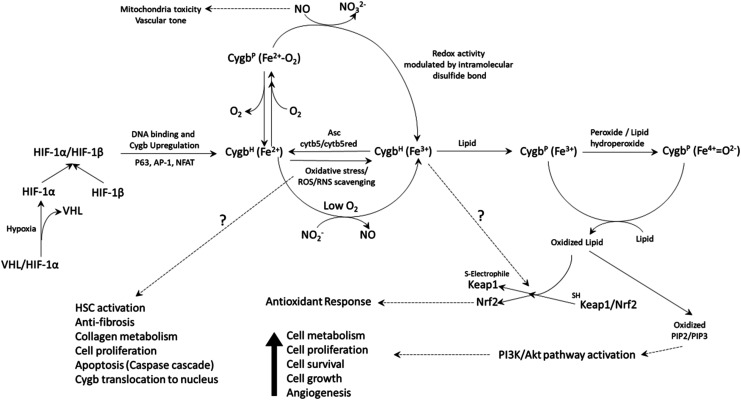
Possible cytoglobin biochemistry and cellular pathways. Cygb is up-regulated by HIF-1 under conditions of hypoxia. Ferrous Cygb^H^ (hexacoordinate) can react with nitrite under hypoxic conditions to generate NO. Under normoxic conditions, the protein binds oxygen and can react with NO to form nitrate. Physiological concentrations of ascorbate, and/or cytochrome b5/cytochrome b5 reductase rapidly return the ferric protein to the ferrous form. Under conditions of stress, lipids can bind to Cygb to generate a pentacoordinate (Cygb^P^) form that (together with an internal disulfide bond) facilitates lipid oxidation reactions. The products of the reaction generate products that can activate numerous cell signalling pathways including the Keap1/Nrf2 antioxidant response (or via a currently unidentified alternative mechanism) and the PI3K/Akt pathway. Dashed lines depict pathways that are presumed or involve multiple steps.

The functions and key molecular mechanisms of Cygb remains unclear. However, emerging evidence supports a multifunctional role of Cygb *in vivo*. Cygb-induced protection against cancer proliferation and hepatic inflammation and fibrinolysis gives us the clearest indication of the role of Cygb in cells, however, the biochemical mechanisms of Cygb in these conditions does not yet have consensus. Increasing bodies of evidence support the redox role of Cygb, be it related to NO homeostasis, ROS scavenging, inhibition of DNA damage or lipid oxidation, it is likely to play a role in the activation of pathways that facilitate cell protection (Figure 2). The role of lipid binding and its alteration of the characteristics of Cygb-induced redox chemistry, together with the observation that Cygb is an efficient lipid peroxidase, has intriguing implications for cell signalling pathways (both in the cytosol and nucleus) and the utilisation of the B2/E9 disulfide as a molecular switch. However, it is clear that these observations require further studies to validate such roles in the proposed physiological and pathological roles of Cygb.

Recent studies using mutations of key functional amino acids have provided insights into the mechanisms of *in vitro* reactions and may provide a way forward to examine specific potential functions of Cygb *in vivo*. For example, the mutation of the E7/B10 amino acids increases/decreases NiR activity by 1000-fold compared with the wild-type [51]. A library of mutations and a comprehensive study of their effects on potential cellular responses would allow specific potential functions to be explored in cell lines and in transgenic animal models.

## Perspectives

- Cygb is a hexacoordinate member of the globin superfamily, discovered in 2001 and ubiquitously expressed in mammalian tissues. Up-regulated by transcription factors such as HIF-1, p63, AP-1 and NFAT under conditions of hypoxia, Cygb appears to play a crucial role in hypoxia response, hepatic fibrosis and progression of specific cancer types.
- Current evidence supports a multifunctional role for Cygb with the oxidation state of a disulfide bond a potential modulator of its redox behaviour. Nitric oxide homeostasis, reactive lipid interaction and ROS scavenging have all been suggested to play a role in the mechanism(s) of cell protection.
- Understanding the roles of the redox chemistry of Cygb and the effect of the disulfide bond as a potential molecular redox switch *in vivo* are crucial for elucidating the mechanisms and pathways that Cygb induces cell protection. Furthermore, a clearer understanding of the molecular mechanisms of Cygb and pathways that Cygb induces would clarify the potential for Cygb as a biomarker or novel target for therapeutic intervention for cancer and other diseases.

## Open Access

Open access for this article was enabled by the participation of University of Essex in an all-inclusive *Read & Publish* agreement with Portland Press and the Biochemical Society under a transformative agreement with JISC.

## Abbreviations

Adgb: androglobin
Cygb: cytoglobin
Cygb^H^: hexacoordinate cytoglobin
Cygb^P^: pentacoordinate cytoglobin
Cyt: cytochrome
DOPA: dioleyl phosphatidic acid (1,2-dioleoyl-*sn*-glycero-3-phosphate)
GbE: globin E
GbX: globin X
Hb: hemoglobin
HDL: high density lipoproteins
HSCs: hepatic stellate cells
LDL: low density lipoproteins
Mb: Myoglobin
Ngb: Neuroglobin
NiR: nitrite reductase
NOD: nitric oxide dioxygenase
PhytoGb: phytoglobin
PIP3 dioleyl phosphatidylinositol 3,4,5-trisphosphate: 1,2-dioleoyl-*sn*-glycero-3-phosphoinositol 3,4,5-trisphosphate
RNS: reactive nitrogen species
ROS: reactive oxygen species
SEC: Size Exclusion Chromatography
SEC-MALLS: size exclusion chromatography multi angle light scattering
STAP: stellate cell activating protein
